# A hybrid machine learning model for pulmonary tuberculosis forecasting of Chongqing with adjacent-region data

**DOI:** 10.1371/journal.pone.0339453

**Published:** 2025-12-31

**Authors:** Yilin Zhang, Hongbo Song, Shuangxueer Zhang, Xiaoying Wang, Junjie Tang

**Affiliations:** 1 State Key Laboratory of Power Transmission Equipment Technology, School of Electrical Engineering, Chongqing University, Chongqing, China; 2 Chongqing University-University of Cincinnati Joint Co-op Institute, Chongqing University, Chongqing, China; 3 College of Engineering, Carnegie Mellon University, Pittsburgh, Pennsylvania, United States of America; 4 Chongqing Medical and Pharmaceutical College, Chongqing, China; University 20 Aout 1955 skikda, Algeria, ALGERIA

## Abstract

Pulmonary Tuberculosis (PTB) remains a serious infectious disease and a major global public health problem. Accurate prediction of PTB epidemics is essential to support health authorities in developing effective prevention and control strategies. This study proposed a novel two-stage hybrid prediction model that integrates a seasonal autoregressive integrated moving average (SARIMA) model and a support vector regression (SVR) model in parallel, followed in series by an extreme learning machine (ELM) optimized via the sparrow search algorithm. Furthermore, recognizing the notable spatial correlation characteristic of airborne PTB transmission, this study incorporates PTB incidence data from surrounding regions of the target area as additional input features to enhance the model with supplementary spatial information, thereby improving prediction accuracy. Validation using real-world PTB incidence data from Chongqing, China, demonstrates the superior performance of the proposed model, which reduces prediction errors by 18.47% to 77.38% compared to existing hybrid models. The inclusion of adjacent regional incidence data further significantly enhances predictive accuracy, reducing errors by 20.92% to 68.74%. The outcomes of this study are expected to facilitate earlier insights into PTB incidence trends and provide valuable support for public health decision-making in PTB prevention and control.

## 1. Introduction

Tuberculosis (TB), an infectious disease caused by *Mycobacterium tuberculosis*, most commonly affects the lungs and is referred to as pulmonary tuberculosis (PTB). As a major global health concern and one of the most persistent diseases in human history, TB continues to pose significant challenges. According to the World Health Organization Global TB Report 2024, an estimated 10.8 million people were suffering from TB worldwide in 2023, resulting in approximately 1.25 million deaths. Between 2020 and 2021, the global TB incidence increased by 3.6%, reversing a previous declining trend of about 2% per year over the past two decades. In 2023, China ranked third globally in the number of new TB cases, accounting for 6.8% of the total burden, behind India (26%) and Indonesia (10%) [1].

Although the Chinese government has implemented a range of interventions, such as the directly observed treatment, short-course (DOTS) strategy [2,3] and a “free” TB diagnosis and treatment policy [4], to strengthen comprehensive TB control, achieving the WHO’s End TB Strategy target of a 90% reduction in TB incidence by 2035 remains a tough challenge. Therefore, there is an urgent need to develop targeted intervention strategies to curb TB transmission and mitigate its public health impact. In this context, building an accurate forecasting model for TB incidence is crucial for obtaining early insights into epidemic trends and facilitating effective TB prevention and control.

In recent years, time series forecasting has been increasingly applied to disease prediction, offering valuable insights for disease management. Accordingly, various single and hybrid models have been employed to forecast tuberculosis (TB) incidence. Commonly used single prediction models for pulmonary TB (PTB) include the seasonal autoregressive integrated moving average (SARIMA) and machine learning approaches such as neural networks and long short-term memory (LSTM).

Studies have shown that the SARIMA model performs well in short-term TB incidence prediction [5–8]. However, such statistical models are often limited by their assumption of linearity and may not always yield satisfactory results. As an alternative, machine learning models, such as recurrent neural networks (RNN), LSTM, backpropagation neural network (BPNN), and support vector regression (SVR), have gained popularity and frequently demonstrate higher predictive accuracy than statistical models [9–12]. Given the complexity of PTB incidence data, hybrid models combining statistical methods with machine learning have been introduced to leverage both linear and nonlinear components of the data, often yielding superior performance. Several studies have indicated that hybrid models, particularly those integrating SARIMA with neural networks, achieve better fitting results and outperform single models [13–20].

Nevertheless, most existing hybrid models adopt simple parallel or series structures with manually configured weights, which can limit predictive accuracy. Moreover, these simplistic architectures present several drawbacks in both construction and application. Their rigid connection patterns restrict adaptability to varied scenarios and hinder the capture of complex data relationships. Although effective in modeling linear trends, such models often struggle with nonlinear mappings and more complicated patterns. They are also prone to overfitting, performing well on training data but generalizing poorly to new data due to oversensitivity to specific training patterns. Additionally, their parameter optimization is often trapped in local optima within complex loss landscapes, leading to suboptimal performance. Therefore, there is a clear need to redesign the structure of hybrid models to enhance the accuracy of TB incidence forecasts.

In predictive modeling, data preprocessing is an essential step that significantly enhances the accuracy of model predictions. Commonly adopted preprocessing techniques include data cleaning, smoothing, standardization, and dataset partitioning. The underlying principle of these methods is to transform raw data into a format suitable for model input. Previous studies have often relied on techniques such as the Kalman Filter and Empirical Mode Decomposition to refine input data [21,22]. While these approaches focus on improving data quality through direct modification, their effectiveness remains highly dependent on the characteristics of the original dataset.

To address this limitation, an alternative strategy involves enriching the input structure rather than merely processing existing data. In this context, parameter optimization algorithms such as the Sparrow Search Algorithm (SSA) are increasingly being applied in disease prediction [23–26]. Introduced by Xue and Shen in 2020 [27], SSA is a swarm intelligence optimization algorithm inspired by the foraging and anti-predation behaviors of sparrows. Compared to traditional optimization methods used in earlier studies [28,29], SSA features fewer hyperparameters, faster convergence, and lower computational cost. It also exhibits a strong global search capability, which helps avoid local optima. These attributes make SSA particularly suitable for tuning parameters in machine learning-based hybrid forecasting models. Nevertheless, to the best of our knowledge, SSA has not yet been employed in the development of prediction models for pulmonary tuberculosis (PTB) incidence.

China comprises 34 provincial-level administrative regions, each exhibiting distinct tuberculosis (TB) epidemic characteristics. Accurate prediction of regional TB trends is therefore essential for formulating targeted prevention and control strategies. As a municipality directly under the central government, Chongqing reported a TB incidence rate falling below 50 per 100,000 for the first time in 2023. Despite this progress, the region remains far from achieving the WHO End TB Strategy target of reducing incidence to below 10 per 100,000. Previous studies have predominantly examined the influence of environmental factors, such as air pollution, meteorological conditions, and sociodemographic variables, on TB incidence in specific areas [30,31].

Nevertheless, PTB persists as a significant infectious respiratory disease. Growing evidence indicates that incidence rates in a given area can be substantially influenced by TB prevalence in its neighboring regions, a phenomenon demonstrated in a township-level study in Taiwan [32]. These findings underscore the critical role of spatial interactions in TB transmission dynamics and highlight the necessity of incorporating spatial dependencies into predictive models. Despite this recognized importance, few studies have explicitly integrated the epidemic data from surrounding areas to refine predictions for a specific target region.

In this study, we aimed to develop a highly accurate prediction model for PTB incidence by redesigning the architecture of a hybrid forecasting framework and incorporating PTB incidence data from adjacent regions. Monthly PTB incidence data from Chongqing and its neighboring and non-adjacent provinces between 2005 and 2019 were utilized. The main work and contributions of this research are summarized as follows:

1) A novel two-stage hybrid model was constructed. In the first stage, a seasonal autoregressive integrated moving average (SARIMA) model and a support vector regression (SVR) model are arranged in parallel. Their outputs are then fed into an extreme learning machine (ELM) model in the second stage. This hierarchical and flexible structure enhances the model’s adaptability to complex incidence patterns.
2) To account for spatial influences, monthly PTB incidence data from adjacent provinces were weighted and incorporated into the ELM model alongside the local incidence data from Chongqing. This integration provides the model with supplementary spatial context, enabling more informed predictions.
3) The SSA was employed to optimize the parameters of the ELM model, improving its predictive performance and generalization ability. The involvement of SSA contributes to achieving more accurate and robust forecasting outcomes.

Through these initiatives, we established an advanced hybrid model to predict PTB incidence in Chongqing, China. It is anticipated that the findings will assist in forecasting future TB trends and support public health authorities in formulating effective prevention and control strategies. Furthermore, the methodology proposed in this study may serve as a reference for constructing prediction models for other infectious diseases.

## 2. Materials and methods

### 2.1 Study area and database

Chongqing, a municipality located in southwestern China, experiences a subtropical monsoon climate characterized by concentrated summer precipitation, resulting in a hot and humid environment. Such warm and humid conditions are considered conducive to the survival and transmission of *Mycobacterium tuberculosis*. Chongqing ranks among the top ten regions in China in terms of tuberculosis infection rates. From 2021 to 2023, the reported annual incidence of PTB in Chongqing was 66.69, 61.7, and 51.7 per 100,000 population, respectively [33].

Data on monthly reported PTB incidence in Chongqing from January 2005 to December 2020 were obtained from the Public Health Science Data Center [34]. The dataset from January 2005 to December 2016 was used as the training set, while data from January 2017 to December 2019 were reserved as the test set for model validation.

### 2.2 Seasonal autoregressive integrated moving average model

The SARIMA model is an advanced statistical model specifically designed for analyzing and forecasting time series data with seasonal characteristics. It extends the standard ARIMA framework by incorporating seasonal components [35]. The ARIMA model itself consists of three core elements: autoregressive (AR), integrated (I), and moving average (MA).

The AR component accounts for temporal dependencies within the time series by using past observations as predictors [36]. Specifically, it models the current value of the series as a linear combination of its previous values plus a noise term. The AR(*p*) model is expressed in equation (1), where *Y*_*t*_, *Y*_*t-*1_, *Y*_*t-*2_, *Y*_*t-p*_ are stationaries and ϕ_0_, ϕ_1_, ϕ_2_, ϕ_*p*_ are constants. *ε*_*t*_ is a Gaussian white noise series with a mean of zero.


$$Y_{t}=\phi_{0}+\phi_{1}Y_{t-1}+\cdot \cdot \cdot +\phi_{p}Y_{t-p}+\varepsilon_{t}=\phi_{0}+\varepsilon_{t}+\sum_{k=1}^{p}\phi_{k}Y_{t-k}$$ (1)

The MA component utilizes a linear combination of historical white noise to predict the present moment through a linear regression model. The MA(*q*) model is expressed in equation (2), where *θ*_1_, *θ*_2_, *θ*_*q*_ are parameters and *ε*_*t*_, *ε*_*t-*1_, *ε*_*t-*2_, *ε*_*t-q*_ are Gaussian white noise series with mean zero.


$$Y_{t}=\varepsilon_{t}-\theta_{1}\varepsilon_{t-1}-\theta_{2}\varepsilon_{t-2}-\cdot \cdot \cdot -\theta_{q}\varepsilon_{t-q}=\varepsilon_{t}-\sum_{k=1}^{q}\theta_{k}\varepsilon_{t-k}$$ (2)

To simplify and comprehend ARIMA models, the backshift operator (*B*) and the difference operator (∇) are used. The backshift operator (*B*) is defined as *B*^*n*^*Y*_*t*_* = Y*_*t-n*_. As for the difference operator, it takes the form ∇^*d*^=(1-*B*)^*d*^, where *d* is the times of differences taken to achieve stationary in the time series data. Hence, equation (1) can be written to equation (3).


$$Y_{t}-\phi_{1}Y_{t-1}-\cdot \cdot \cdot -\phi_{p}Y_{t-p}=Y_{t}-\sum_{k=1}^{p}\phi_{k}Y_{t-k}=\phi_{0}+\varepsilon_{t}=\phi (B)Y_{t}$$ (3)

where ϕ(B) is the autoregression polynomial of order *p*, defined by:


$$\phi (B)=1-\phi_{1}B-\phi_{2}B^{2}-\cdots -\phi_{p}B^{p}$$ (4)

Equation (2) can be written to equation (5).


$$Y_{t}=\varepsilon_{t}-\theta_{1}\varepsilon_{t-1}-\cdot \cdot \cdot -\theta_{q}\varepsilon_{t-q}=\varepsilon_{t}-\sum_{k=1}^{q}\theta_{k}\varepsilon_{t-k}=\theta (B)\varepsilon_{t}$$ (5)

where *θ*(*B*) is the moving average polynomial of order *q*, defined by:


$$\theta (B)=1-\theta_{1}B-\theta_{2}B^{2}-\cdot \cdot \cdot -\theta_{q}B^{q}$$ (6)

Therefore, the ARIMA (*p*, *d*, *q*) model can be represented by using the backshift and difference operators. The equation of the ARIMA model is shown below:


$$\phi (B)\nabla^{d}Y_{t}=\theta (B)\varepsilon_{t}$$ (7)

SARIMA can be considered an extension of the ARIMA [37]. It expands the ARIMA model by merging three additional parameters to define the seasonal components of the ARIMA model. The parameters of the SARIMA are denoted as SARIMA (*p, d, q*) (*P, D, Q, s*). The non-seasonal components (*p, d, q*) remain the same as the corresponding ARIMA components. The seasonal components (*P, D, Q*) introduce additional specific to the seasonal behavior of the time series data, and *s* indicates the periodicity or seasonality of the data.

The SARIMA model takes the seasonal factors into account, then the seasonal difference operator is defined as ∇_*S*_^*D*^=(1*-B*^*S*^)^*D*^. According to the autoregression polynomial *ɸ*(*B*) and the moving average polynomial *θ*(*B*), the seasonal autoregression and moving average polynomials are defined in equations (8)–(10).


$$\phi_{s}(B)=1-\phi_{1}B^{s}-\phi_{2}B^{2s}-\cdots -\phi_{p}B^{ps}$$ (8)

$$\theta_{s}(B)=1-\theta_{1}B^{s}-\theta_{2}B^{2s}-\cdots -\theta_{q}B^{qs}$$ (9)

$$\phi (B)\phi_{s}(B)\nabla^{d}\nabla_{s}^{D}Y_{t}=\theta (B)\theta_{s}(B)\varepsilon_{t}$$ (10)

### 2.3 Support vector regression model

SVR is a machine learning algorithm specifically designed for regression analysis [38], widely used to model and predict continuous outcomes. The core objective of SVR is to identify a regression function that minimizes the error between predicted and actual values. In doing so, it seeks to maximize the margin around the fitted function where errors are tolerated, thereby enhancing generalization capability. The SVR function *f*(**x**) is shown in equation (11):


$$f(𝐱)={𝐰}^{T}\varphi (𝐱)+b$$ (11)

where *φ*(**x**) is the feature space obtained by mapping the input *x* through a kernel function, **w** is the weight vector of the model, and *b* is the bias. If the training sample falls within this interval band, it can be considered a correct prediction. Therefore, the penalty function of SVR is:


$$R(C)=\underset{w,b}{\min}\frac{\text{1}}{\text{2}}{\left\|𝐰\right\|}^{2}+C\sum_{i=1}^{m}\left(\xi_{i}+\xi_{i}^{*}\right)$$ (12)

Under constraints:


$$f({𝐱}_{i})-{𝐲}_{i}\leq \varepsilon +\xi_{i}$$ (13)

$${𝐲}_{i}-f({𝐱}_{i})\leq \varepsilon +\xi_{i}^{*}$$ (14)

$$\xi_{i}\geq 0,\xi_{i}^{*}\geq 0,i=1,2,...,m$$ (15)

where *C* is the regularization parameter, *ε* is the insensitive loss factor, and *m* is the number of samples. The training error above *ε* is *ξ*_*i*_, while the training error under *ε* is *ξ*_i_^*^.

After solving the quadratic optimization problem with inequality constraints, the weight vector **w** is computed as shown in equation (16). The parameters *α*_i_^*^ and *α*_*i*_ are Lagrangian multipliers.


$$𝐰=\sum_{i=1}^{N}\left(\alpha_{i}^{*}-\alpha_{i})\varphi ({𝐱}_{i}\right)$$ (16)

Finally, the SVR regression function is derived as the equation shown in equation (17).


$$f(𝐱)=\sum_{i=1}^{N}\left(\alpha_{i}^{*}-\alpha_{i})K({𝐱}_{i},𝐱\right)+b$$ (17)

where *K*(**x**_i_, **x**_j_) is the kernel function. The kernel function can be computed by the inner product of **x**_i_ and **x**_j_. In the feature space, *K*(**x**_i_, **x**_j_) = *φ*(**x**_i_)·*φ*(**x**_j_).

### 2.4 Extreme learning machine model

ELM is a simple and effective algorithm that is designed for training single hidden layer feed-forward neural networks (SLFNs). The architecture of an SLFN can be described by a triple (*d, m, k*), where *d* is the dimensionality of input data, *m* is the number of hidden nodes, and *k* is the number of classes of input data. Given a training set:


$$D=\left\{\left(x_{i},y_{i}\right)|x_{i}\in R^{d},y_{i}\in R^{k}\right\},1\leq i\leq n$$ (18)

The output function *F*(*x*) of the SLFNs can be expressed as:


$$F(x_{i})=\sum_{j=1}^{m}\beta_{j}g(w_{j}\cdot x_{i}+b_{j})$$ (19)

where *β*_*j*_ is the weight vector connecting the *j*^*th*^ hidden node with the output nodes, while *w*_*j*_ is the weight vector connecting the *j*^*th*^ hidden node with the input nodes. Moreover, *b*_*j*_ is the bias parameter of the *j*^*th*^ hidden node, and g(•) is the activation function.

Among the parameters above, *w*_*j*_ and *b*_*j*_ are randomly selected. *β*_*j*_ can be determined from the linear system shown below:


$$\sum_{j=1}^{m}\beta_{j}g(w_{j}\cdot x_{i}+b_{j})=y_{i}$$ (20)

The linear system can also be written as matrix form, **Y = Hβ**. In the matrix formula, **H** is the output matrix of the input layer of SLFN, which is usually a non-square matrix. The expansion of **H**, **β**, and **Y** can be represented as:


$$H=\left[\begin{array}{ccc} {}*20cg(w_{1}\cdot x_{1}+b_{1}) & \cdots & g(w_{m}\cdot x_{1}+b_{m})\\ \vdots & \vdots & \vdots \\ g(w_{1}\cdot x_{n}+b_{1}) & \cdots & g(w_{m}\cdot x_{n}+b_{m}) \end{array}\right]$$ (21)

$$\begin{array}{c} \beta ={\left[\beta_{1}^{T},\beta_{2}^{T},\cdots ,\beta_{m}^{T}\right]}^{T},Y={\left[y_{1}^{T},y_{2}^{T},\cdots ,y_{n}^{T}\right]}^{T} \end{array}$$ (22)

### 2.5 Spatial correlation analysis

To analyze the nationwide incidence of PTB across provinces and cities, the monthly PTB incidence in the target area served as the primary training dataset. Given that the time series comprises only 192 data points, the limited sample size may constrain prediction accuracy. To address this, we augmented the input data by incorporating monthly incidence rates from adjacent areas in the spatial dimension, thereby increasing the effective information available for modeling. The data from different regions were combined using weights derived from spatial autocorrelation analysis. Furthermore, as PTB is a severe respiratory infectious disease, its incidence is likely to exhibit geographical dependence, making spatial correlation analysis a valuable component for improving prediction accuracy.

Spatial autocorrelation analysis allows for the assessment of whether incidence rates in surrounding regions are correlated with those in the target area. Since spatial patterns may vary across the study area, local spatial autocorrelation analysis was employed to examine region-specific distribution characteristics. The degree of spatial association was quantified using Moran’s Index (Moran’s I) [39]. Regions exhibiting stronger spatial correlation with the target area were assigned higher weights in the prediction model.

The local Moran’s Index can be determined by using the formula shown in equation (23):


$$I_{i}=\frac{x_{i}-\bar{x}}{S^{2}}\sum_{j=1}^{n}W_{ij}(x_{j}-\bar{x}),i\neq j$$ (23)

where *n* indicates the number of regions covered in the study, *x*_*i*_ (*x*_*j*_) represents the incidence of tuberculosis in region *i* (*j*), $\overset{―}{x}$ denotes the average incidence of tuberculosis across the study area, *S*^2^ is the variance, and *W*_*ij*_ is the element in row *i* and column *j* of the spatial weight matrix.

The value range of Moran’s Index is [−1, 1]. The extent of correlation between different regions can be divided into three conditions based on the value of Moran’s Index:

1) A value greater than 0 (or close to 1) indicates positive spatial correlation.
2) A value less than 0 (or close to −1) indicates negative spatial correlation.
3) A value near 0 suggests weak or no spatial correlation.

As spatial autocorrelation analysis is grounded in probability theory, it is essential to evaluate the statistical significance of the results. For local spatial autocorrelation, a Z-test is commonly applied to the statistic *I*_*i*_, with p < 0.05 indicating significant local spatial autocorrelation.

**Fig 1:**
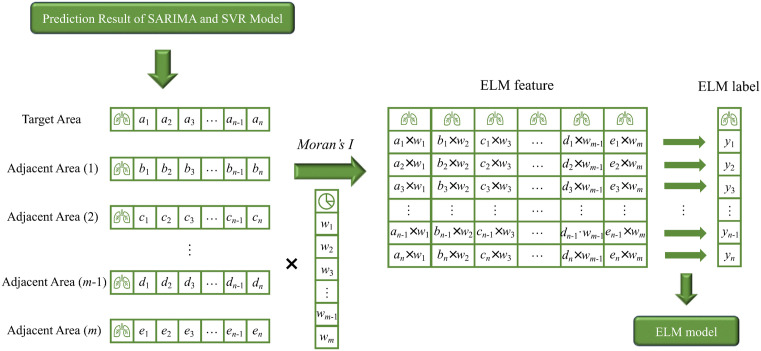
The schematic diagram of merging the data of adjacent areas.

After computing Moran’s Index between regions, PTB incidence data from areas showing high spatial correlation with the target region were included as additional input features, as illustrated in Fig 1.

### 2.6 The sparrow search algorithm of the ELM model

The SSA is a swarm intelligence-based optimization technique inspired by the foraging and anti-predatory behaviors of sparrows. In SSA, each sparrow represents a potential solution within the search space. The algorithm iteratively updates the position of each sparrow by combining individual experience with collective intelligence, guiding the population toward optimal regions.

When integrated with the ELM, SSA effectively optimizes the input weights *w*^(*j*)^ and biases *b*^(*j*)^ of the hidden layers. In conventional ELM, these parameters are typically initialized randomly, which can lead to suboptimal performance. By employing SSA, these values are systematically refined through an iterative process wherein each sparrow’s position corresponds to a candidate set of parameters. During each iteration, the fitness value—representing the performance of the objective function for each candidate solution—is evaluated to guide the search direction. This approach enhances the ELM’s ability to converge to a more effective and generalized model configuration.


$$𝐗=\left[\begin{array}{cccc} {}*20cx_{11} & x_{12} & \cdots & x_{1n}\\ x_{21} & x_{22} & \cdots & x_{2n}\\ \vdots & \vdots & \ddots & \vdots \\ x_{m1} & x_{m2} & \cdots & x_{mn} \end{array}\right]$$ (24)

$${𝐅}_{𝐗}=\left[\begin{array}{c} {}*20cf([x_{11},x_{12},\cdots ,x_{1n}])\\ f([x_{21},x_{22},\cdots ,x_{2n}])\\ \vdots \\ f([x_{m1},x_{m2},\cdots ,x_{mn}]) \end{array}\right]$$ (25)

The matrix **X** is the position of a group of sparrows, and each row of **X** indicates a feasible solution. Specifically, **X** represents the vectors consisting of *w*^(*j*)^ and *b*^(*j*)^ (0* < j < k + *1). *m* is the number of sparrows, and *n* represents the number of values to be optimized. Each row of **F**_**X**_ denotes the fitness value corresponding to each sparrow.

The best individuals within the group are given priority for food during the search process. As explorers, they have access to a larger foraging range than their followers. Within each iteration, the locations of producers are updated as below:


$$X_{i,j}^{t+1}=\{\begin{array}{cc} {}*20cX_{i,j}^{t}\cdot \exp(\frac{-i}{\alpha \cdot iter_{\max}}) & R_{2}<ST\\ X_{i,j}^{t}+Q\cdot L & R_{2}\geq ST \end{array}$$ (26)

where *t* is the current iteration number; *X*^*t*^_*i,j*_ is the *j*^*th*^ variable of the *i*^*th*^ sparrow at iteration *t*. The *iter*_*max*_ represents the maximum iteration number; *α* is a random number located in [0,1]; *R*_2_ (*R*_2_∈[0,1]) and ST (ST∈[0.5,1]) are warning values and safety threshold, respectively. Moreover, *Q* is a random number following a normal distribution; *L* is a 1 × *d* matrix where every element is 1.

When *R*_2_ *< ST*, this means that there are no predators around and the explorers are allowed to conduct a global search. Conversely, when *R*_2_* ≥ ST*, it implies that some sparrows have spotted predators, and all the sparrows need to take action. Within each iteration, the updating rule of a scrounger’s location is described as follows:


$$X_{i,j}^{t+1}=\{\begin{array}{cc} {}*20cQ\cdot \exp(\frac{X_{worst}^{t}-X_{i,j}^{t}}{i^{2}}) & i>\frac{n}{\text{2}}\\ X_{p}^{t+1}+|X_{i,j}^{t}-X_{p}^{t+1}|\cdot \left(A^{T}\cdot {\left(A\cdot A^{T}\right)}^{-1}\right)\cdot L & i\leq \frac{n}{\text{2}} \end{array}$$ (27)

where *X*_*p*_ is the optimal position held by producers and *X*^*t*^_*worst*_ is the worst position at the current iteration. *A* is a 1 × *d* vector, and each element of *A* is randomly set to 1 or −1.

When *i > n/*2, the *i* follower with a lower fitness value is in poor condition and needs to fly elsewhere to feed. In SSA, the initial locations of individuals who are aware of danger are randomly generated in the population. The updating function for a sparrow realizing danger can be written as:


$$X_{i,j}^{t+1}=\{\begin{array}{cc} {}*20cX_{best}^{t}+\beta \cdot |X_{i,j}^{t}-X_{best}^{t}| & f_{i}\neq f_{g}\\ X_{i,j}^{t}+K\cdot \frac{\left|X_{i,j}^{t}-X_{worst}^{t}\right|}{(f_{i}-f_{w})+\varepsilon } & f_{i}=f_{g} \end{array}$$ (28)

where *X*^*t*^_*best*_ is the current optimal position; *β* is the control parameter of step size, following a standard normal distribution. *K* represents the direction of the sparrow’s movement and is also a step control parameter, and it is a random number within the interval [−1, 1]; moreover, ε is a constant to avoid a zero-denominator; *f*_*i*_, *f*_*g*_, and *f*_*w*_ are the present sparrow’s fitness value, current global best fitness values, and worst fitness values, correspondingly.

When *f*_*i*_ ≠* f*_*g*_, the sparrow is at the edge of the group. Additionally, when *f*_*i*_ =* f*_*g*_, the sparrow in the middle of the group is aware of the danger and needs to stay close to other sparrows to avoid being preyed upon.

### 2.7 Hybrid SARIMA-SVR-ELM model

The SARIMA model is primarily employed to extract and analyze linear patterns in time series data, while the SVR model excels in handling nonlinear and high-dimensional prediction tasks. In practical applications, time series data often contain both linear and nonlinear components. By integrating SARIMA and SVR in a parallel configuration, the hybrid model effectively captures both types of patterns, thereby achieving superior performance compared to individual models. In the proposed framework, the parallel SARIMA-SVR structure constitutes the first stage, and its outputs are fed into an optimized ELM model in the second stage, forming a series-connected two-stage forecasting system.

Monthly PTB incidence from 2005–2016 was used for training, and 2017–2019 was reserved for evaluation. SARIMA and SVR were fitted on the training period and then used to produce one-step-ahead rolling forecasts for each month across the entire study horizon (2005–2019) for the target region and its adjacent regions. For each month, the resulting 2 × 6 base-model forecasts were concatenated as meta-features. An extreme learning machine (ELM) was trained on the 2005–2016 meta-features to predict the target region’s incidence (with the observed incidence as the meta-target), and its parameters were optimized via the sparrow search algorithm (SSA). Performance was assessed from 2017 to 2019 by comparing the ELM’s out-of-sample predictions with the observed monthly incidence in the target region.

**Fig 2:**
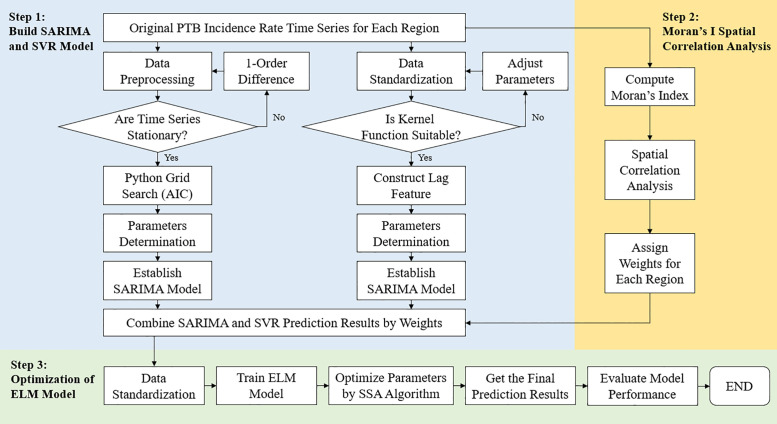
The flow chart of the proposed hybrid model.

**Fig 3:**
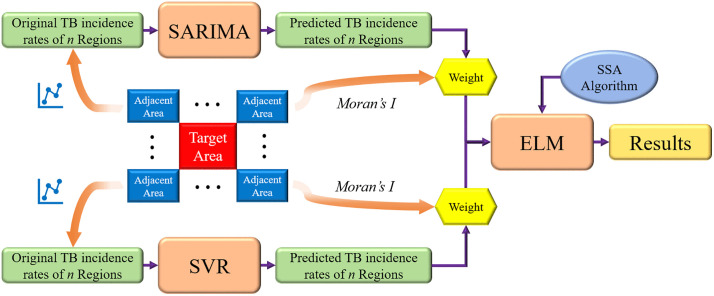
The schematic diagram of the proposed hybrid model.

The flowchart and schematic diagram of the proposed hybrid model are presented in Figs 2 and 3, respectively.

To evaluate predictive performance, three metrics were employed: Mean Absolute Error (MAE), Root Mean Squared Error (RMSE), and Mean Absolute Percentage Error (MAPE) [13]. The formulas for these metrics are provided below.


$$MAE=\frac{\text{1}}{n}\sum_{i=1}^{n}\left|Y_{i}-{\hat{Y}}_{i}\right|$$ (29)

$$RMSE=\sqrt{\frac{\text{1}}{n}{\sum_{i=1}^{n}\left(Y_{i}-{\hat{Y}}_{i}\right)}^{2}}$$ (30)

$$MAPE=\frac{100\%}{n}\sum_{i=1}^{n}\left|\frac{Y_{i}-{\hat{Y}}_{i}}{Y_{i}}\right|$$ (31)

where *Y*_*i*_ is the real incidence rate at time *i* in the test set, *Ŷ*_*i*_ is the estimated incidence rate at time *i* in the test set*,* and *n* represents the number of predictions in the test set.

**Fig 4:**
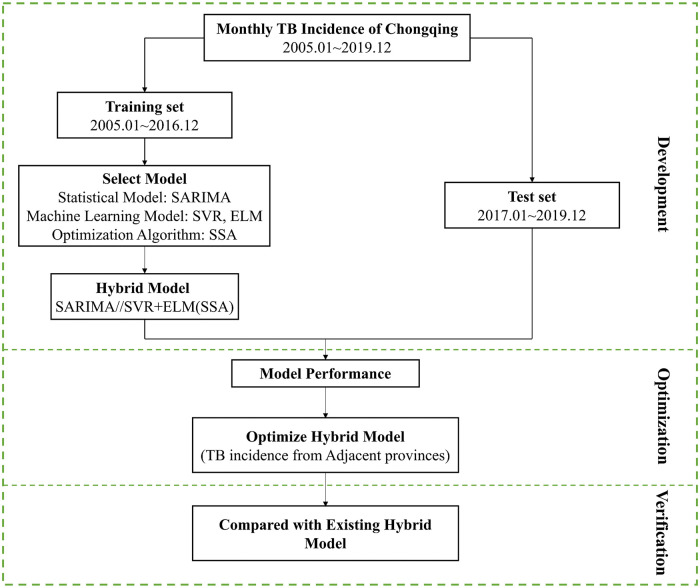
The flowchart of the study.

All analytical procedures, including time series data extraction and analysis, construction of the SARIMA, SVR, and ELM models, and spatial autocorrelation analysis, were performed using Python 3.9.10 [40]. The overall workflow of the study is illustrated in Fig 4.

## 3. Case studies

In this section, a series of baseline comparisons and ablation studies are conducted to verify the effectiveness of the proposed method. Data from 2005 to 2016 are used for training, and data from 2017 to 2019 are used for testing. The input features of the model consist of the historical tuberculosis incidence rates of Chongqing and its surrounding areas, and the lookback window length is set to 12. The three error metrics, MAE, RMSE, and MAPE, described in Section 2.7, are adopted as the evaluation criteria.

### 3.1 Time series characteristics of PTB incidence in Chongqing

**Fig 5:**
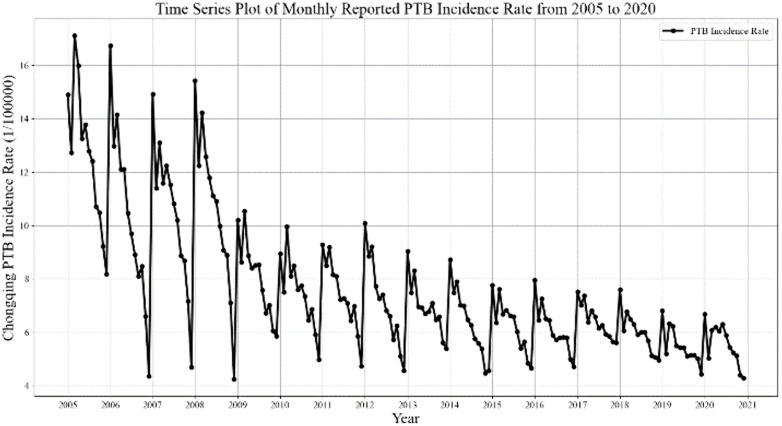
Time series plot of monthly reported PTB incidence in Chongqing from 2005 to 2020.

Fig 5 displays the time series of reported PTB incidence in Chongqing from January 2005 to December 2020. The series exhibits a combination of linear and nonlinear components, along with a clear long-term decreasing trend and pronounced seasonal fluctuations. Annually, two distinct peaks are observed in January and March, a pattern that aligns with findings from previous studies [10,41,42]. Data from the year 2020 were excluded from model development and testing due to potential disruptions in TB reporting caused by the COVID-19 pandemic.

**Fig 6:**
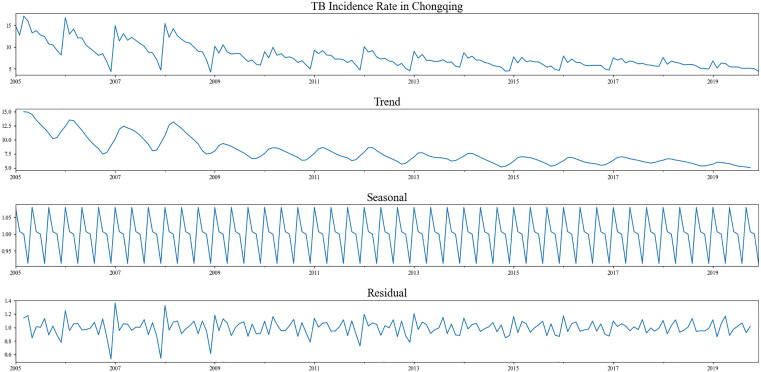
The seasonal decomposition outcome of PTB incidence time series in Chongqing from 2005 to 2020.

The PTB incidence series exhibits an apparent seasonal pattern that fluctuates periodically over the course of a year. This periodicity was confirmed through seasonal decomposition, which revealed a more clearly defined cyclical component, as shown in Fig 6.

### 3.2 Performance of the proposed model compared to simple baseline models

**Fig 7:**
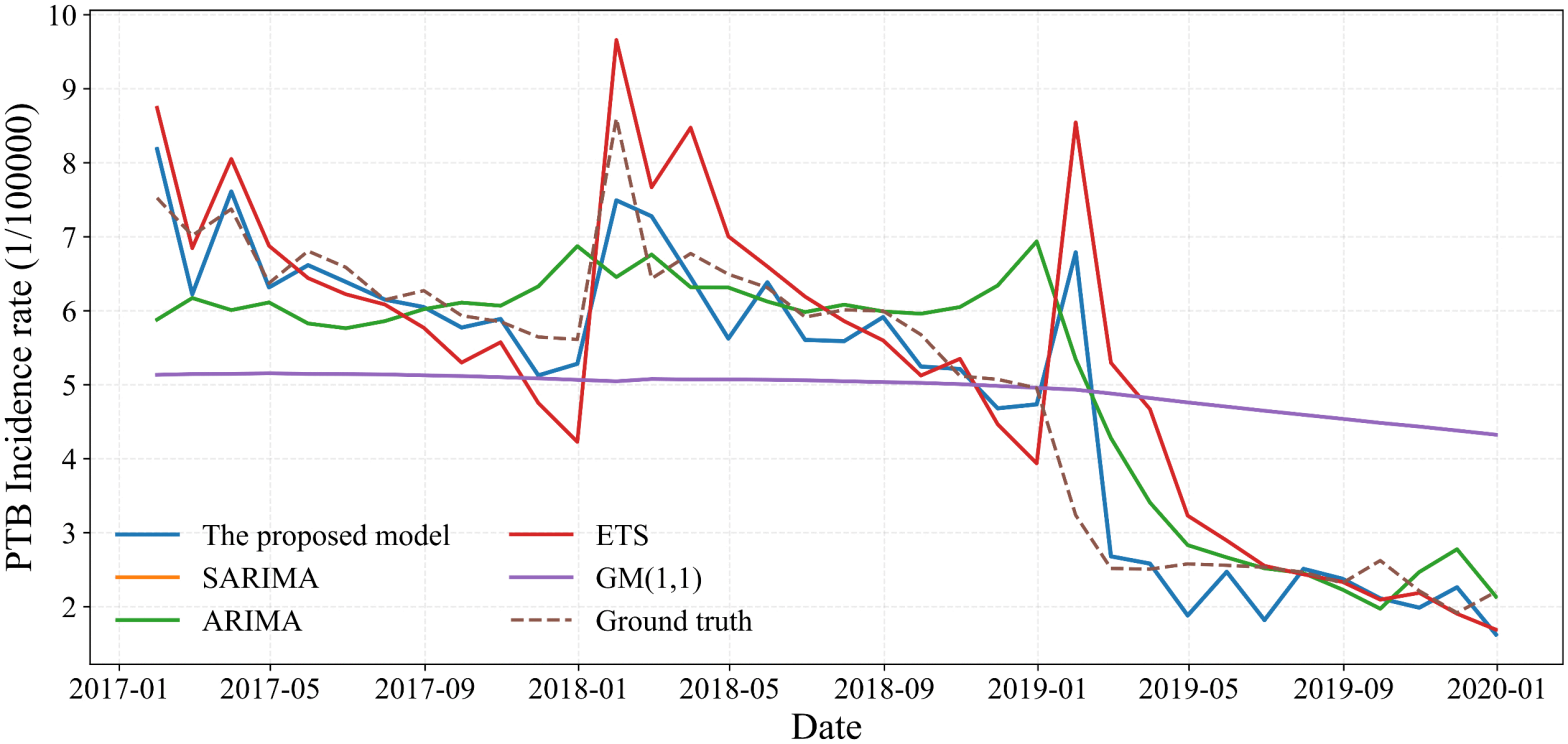
Comparison of the hybrid model with existing statistical models.

**Fig 8:**
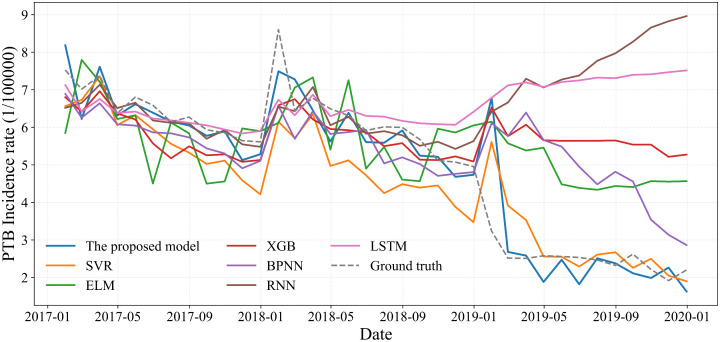
Comparison of the hybrid model with a machine learning model.

To evaluate the effectiveness of the proposed hybrid model, its performance was benchmarked against a range of statistical and machine learning models. The statistical counterparts included SARIMA, ARIMA, grey model first order one variable (GM(1,1)), and the error, trend, seasonality (ETS) method. The machine learning models comprised SVR, ELM, SSA-optimized ELM (ELM(SSA)), XGBoost, BPNN, RNN, generalized regression neural network (GRNN), autoregressive neural network (ARNN), and LSTM. A comparative summary of their performance is provided in Table 1 and Figs 7 and 8. Among the statistical models, SARIMA achieved the lowest prediction errors. For machine learning models, XGBoost and LSTM demonstrated superior accuracy in capturing nonlinear trends. Notably, the standard ELM model offered the fastest computational speed, and its predictive accuracy was further enhanced through optimization with the SSA algorithm.

**Table 1:** Comparison of the proposed hybrid model with the existing simple models.

Models	MAE	RMSE	MAPE (%)
Statistical models			
SARIMA [8]	0.660	0.919	15.008
ARIMA [43]	0.708	0.979	15.497
GM (1,1) [41]	1.510	1.705	41.503
ETS [44]	0.763	1.242	19.107
Machine learning models			
SVR [19]	0.844	1.048	17.656
ELM	1.395	1.657	42.053
XGBoost [45]	1.431	1.934	49.394
BPNN [11]	1.185	1.561	37.318
RNN [46]	1.968	3.117	76.536
LSTM [13]	1.881	2.821	70.785
**The proposed model**	**0.434**	**0.737**	**10.795**

Although the standalone performance of the SVR model was surpassed by XGBoost and LSTM in our tests, it remains well-suited for modeling nonlinear trends in time series, particularly with limited datasets. The robustness of SVR stems from its use of an ε-insensitive band around the regression function, which excludes samples within this margin from the loss calculation. This mechanism confers a high tolerance to noise and outliers, enabling SVR to achieve reliable generalization even on small sample sizes. It is for this key reason that SVR was selected as a component in our hybrid forecasting framework.

### 3.3 Ablation study on different hybrid model configurations

To identify the optimal hybrid forecasting structure, we systematically evaluated the performance of various model combinations. This involved testing hybrid frameworks comprising three different models—specifically, SARIMA combined with either SVR, XGBoost, or BPNN, and subsequently integrated with ELM—under different connection architectures. The performance metrics of all candidate hybrid models are summarized in Table 2.

**Table 2:** Prediction performance of PTB incidence by using different forms of hybrid models.

Models	MAE	RMSE	MAPE (%)
SARIMA, SVR, and ELM Model			
SARIMA+SVR + ELM	1.270	1.900	46.156
SARIMA+SVR + ELM(SSA)	1.272	1.899	46.216
SARIMA//SVR//ELM	0.701	1.170	24.182
SARIMA//SVR//ELM (SSA)	0.612	1.070	17.385
SARIMA//SVR + ELM	0.768	1.011	22.867
**SARIMA//SVR + ELM(SSA)**	**0.434**	**0.737**	**10.795**
SARIMA, XGBoost, and ELM Model			
SARIMA+XGBoost+ELM	1.562	2.205	55.320
SARIMA+XGBoost+ELM(SSA)	1.562	2.208	55.382
SARIMA//XGBoost//ELM	0.894	1.329	31.624
SARIMA//XGBoost//ELM(SSA)	0.896	1.247	29.194
SARIMA//XGBoost+ELM	0.979	1.451	34.582
**SARIMA//XGBoost+ELM(SSA)**	0.849	1.321	25.443
SARIMA, BPNN, and ELM Model			
SARIMA+BPNN+ELM	1.117	1.397	32.080
SARIMA+BPNN+ELM(SSA)	1.141	1.419	33.648
SARIMA//BPNN//ELM	0.991	1.206	28.338
SARIMA//BPNN//ELM(SSA)	0.725	1.060	20.367
SARIMA//BPNN+ELM	0.948	1.186	28.508
**SARIMA//BPNN+ELM(SSA)**	0.742	1.287	18.194

As indicated by the results in Table 2, the two-stage hybrid model labeled SARIMA//SVR + ELM(SSA) achieved the lowest prediction errors, demonstrating superior performance over other hybrid configurations. Moreover, models employing the SSA consistently showed improved accuracy compared to their non-optimized counterparts, confirming that SSA effectively optimizes ELM parameters and enhances predictive performance. These findings are visually supported in , where the calibration curve of the SARIMA//SVR + ELM(SSA) model closely aligns with the reference line, indicating a high level of predictive accuracy. Based on these comprehensive results, the SARIMA//SVR + ELM(SSA) structure was selected as the final model for PTB incidence forecasting.

**Fig 9:**
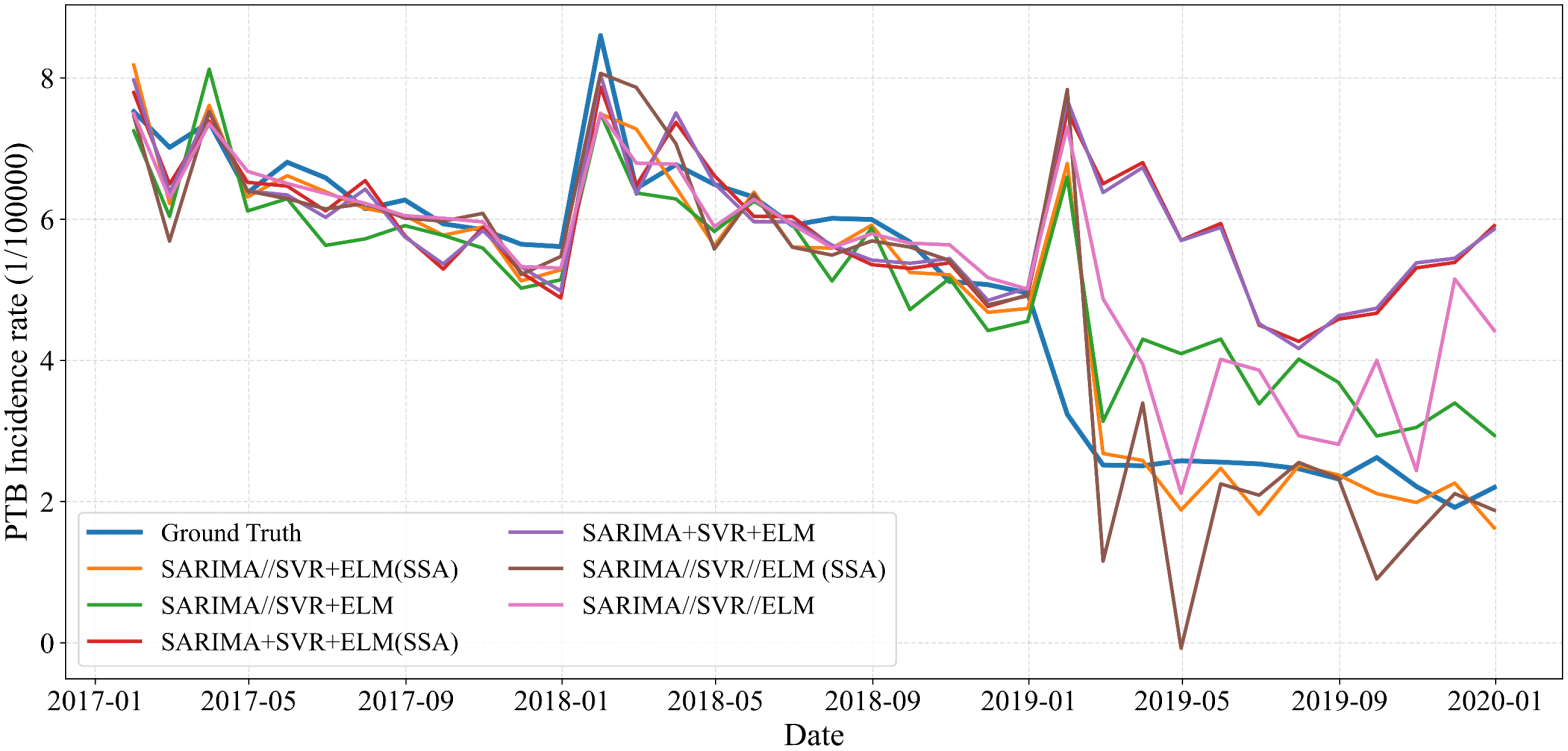
Performance of SARIMA, SVR, and ELM model in predicting PTB incidence in Chongqing.

**Fig 10:**
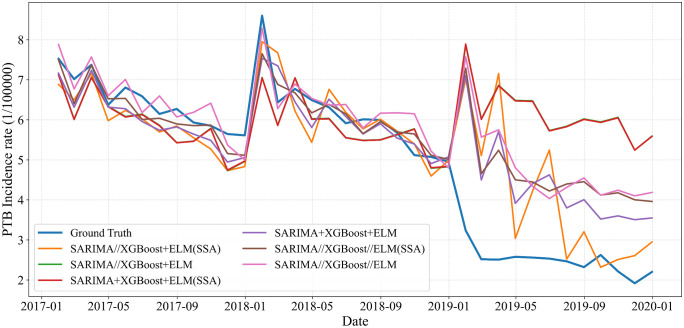
Performance of SARIMA, XGBoost, and ELM model in predicting PTB incidence in Chongqing.

**Fig 11:**
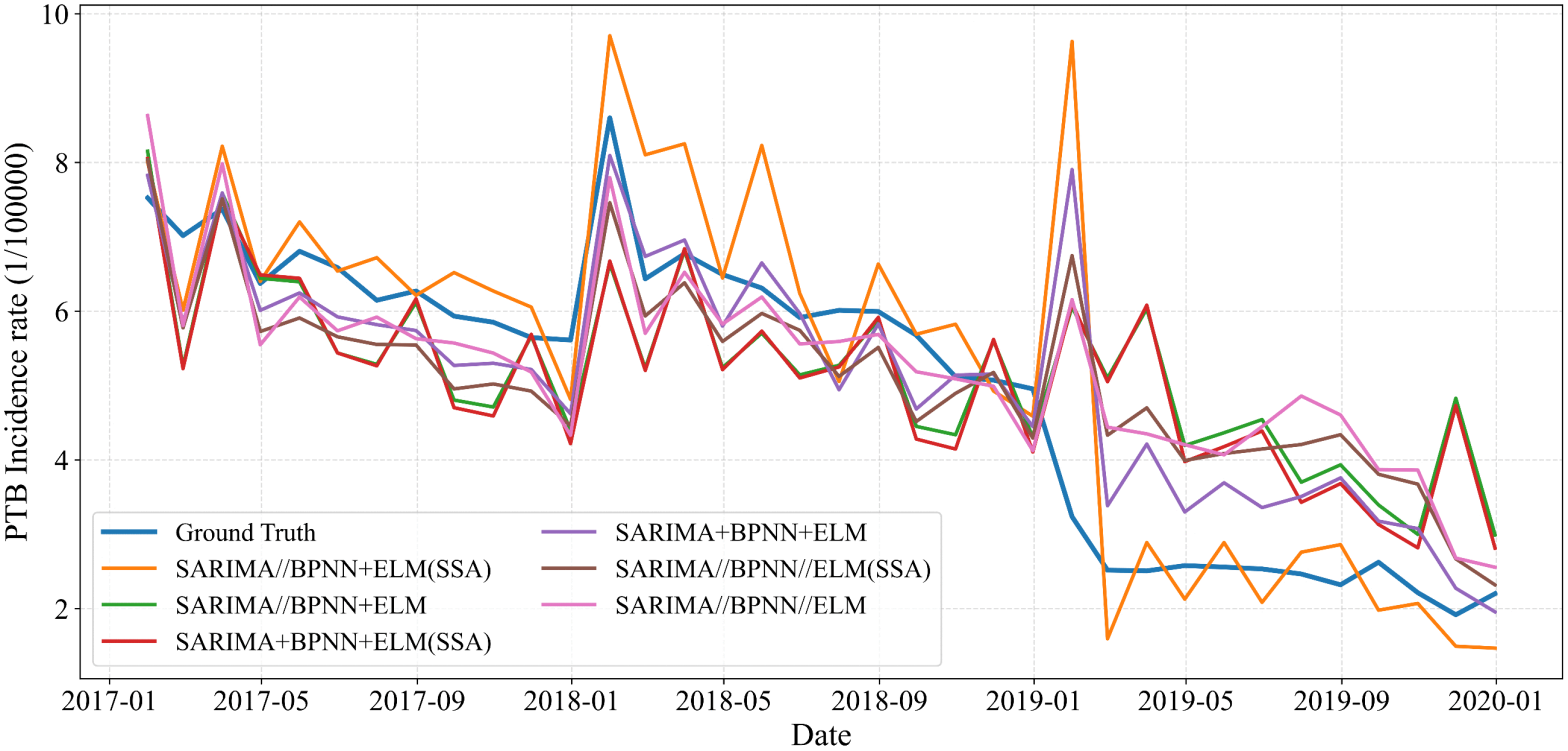
Performance of SARIMA, BPNN, and ELM model in predicting PTB incidence in Chongqing.

### 3.4 Ablation study on the surrounding area of PTB incidence features

**Fig 12:**
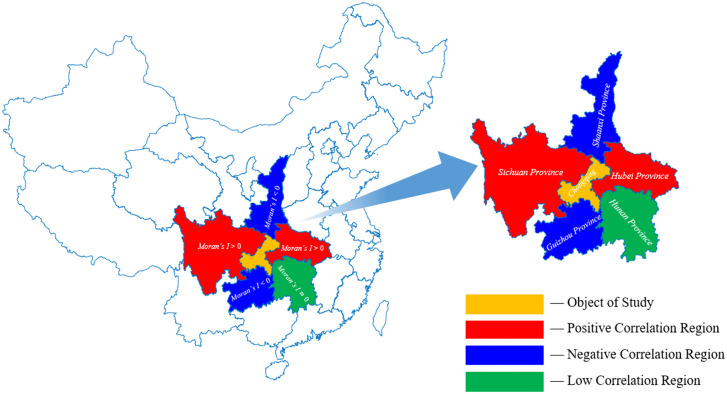
The schematic diagram of the spatial correlation analysis of Chongqing and its five adjacent provinces. Note: The base map layer is derived from public domain vector data provided by Natural Earth (http://www.naturalearthdata.com).

**Fig 13:**
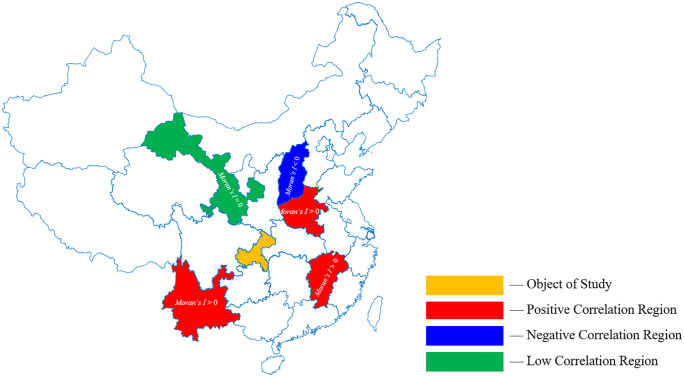
The schematic diagram of the spatial correlation analysis of Chongqing and its five non-adjacent provinces. Note: The base map layer is derived from public domain vector data provided by Natural Earth (http://www.naturalearthdata.com).

To enhance the predictive accuracy of the hybrid model, we investigate whether PTB incidence in surrounding regions influences the prediction of PTB incidence in Chongqing. First, Moran’s index is calculated to assess the spatial correlation between Chongqing and five adjacent provinces (Hubei, Shaanxi, Sichuan, Guizhou, Hunan) as well as five non-adjacent provinces (Shanxi, Henan, Jiangxi, Yunnan, Gansu). A schematic diagram of Moran’s index is presented in Figs 12 and 13.

The effect of incorporating different numbers of adjacent provinces as auxiliary predictors for Chongqing is evaluated. The results, summarized in Table 3, indicate that including geographically proximate information consistently improves prediction performance. Compared to using data from Chongqing alone (0 provinces; MAE = 0.989, RMSE = 1.476, MAPE = 34.53%), the addition of neighboring provinces leads to a steady reduction in prediction error, with the best performance achieved when all five adjacent provinces are included (MAE = 0.434, RMSE = 0.737, MAPE = 10.80%). This pattern aligns with the notion of spatial spillover effects and shared epidemiological dynamics: PTB incidence in neighboring regions serves as a valuable predictor for Chongqing, and integrating these external signals into the feature set effectively enhances forecasting accuracy.

**Table 3:** Comparison of the prediction performance after adding the PTB incidence data from adjacent or non-adjacent regions.

Models	MAE	RMSE	MAPE (%)
Adding adjacent region data (Hubei, Shaanxi, Guizhou, Sichuan, Hunan)			
0 Province	0.989	1.476	34.534
1 Province	0.582	1.021	15.596
2 Provinces	0.549	0.946	16.256
3 Provinces	0.647	1.128	17.955
4 Provinces	0.581	0.932	16.972
**5 Provinces**	**0.434**	**0.737**	**10.795**
Adding non-adjacent region data	1.015	1.432	34.704

However, the performance improvement is not strictly monotonic at each incremental step—for instance, the model with three provinces performs slightly worse than that with two. Such fluctuations are common in multivariate time-series modeling, where nonlinear feature interactions, partial redundancy or collinearity among predictors, and the inherent bias–variance trade-off with increasing input dimensions can lead to minor deviations, even amid a clear overall downward trend in error.

**Fig 14:**
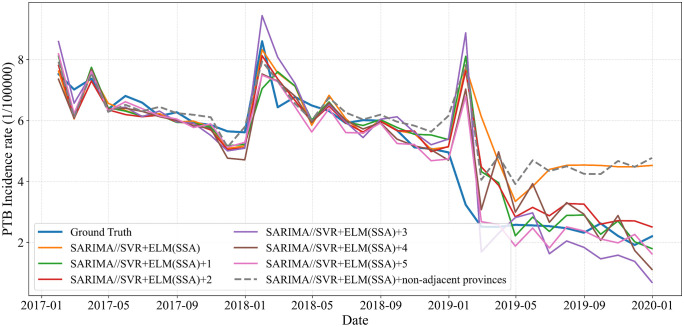
Forecasting curves after adding data from adjacent or non-adjacent provinces.

Furthermore, incorporating data from non-adjacent provinces leads to a decline in performance (MAE = 1.015, RMSE = 1.432, MAPE = 34.70%), suggesting that these variables contribute little meaningful information and instead introduce noise or distributional discrepancies. The details are shown in Fig 14. In summary, while adjacent provinces offer valuable contextual signals that enhance prediction accuracy, non-adjacent provinces tend to act as uninformative or confounding features and should be excluded unless justified by strong domain-specific relevance.

### 3.5 Performance of the proposed model compared to hybrid baseline models

**Fig 15:**
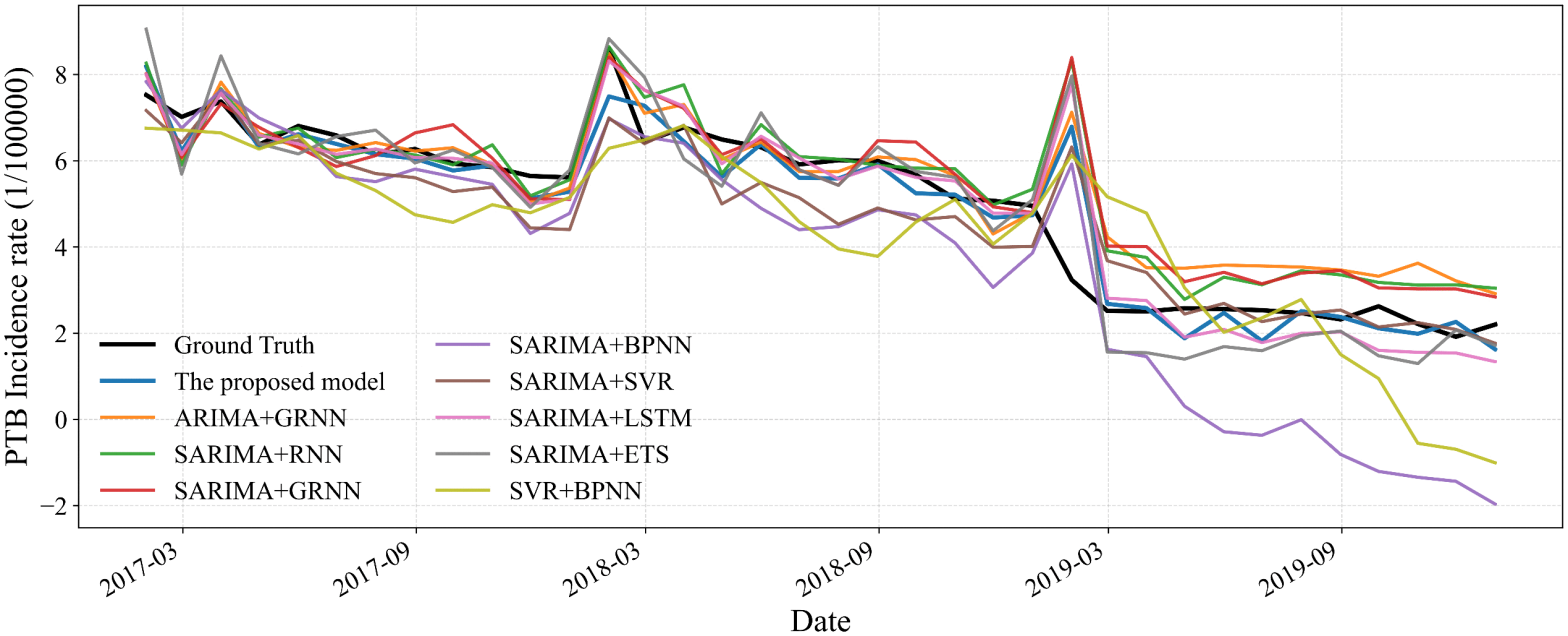
Forecasting curves of the hybrid model with existing hybrid models.

In previous studies, various hybrid models have been developed to predict the incidence of PTB. To evaluate the performance of our proposed hybrid model, we compared it against several existing hybrid models reported in the literature. As summarized in Table 4 and visualized in Fig 15, the proposed model achieves the lowest prediction errors on the test datasets. Specifically, it reduces prediction error by 18.47% to 77.38% compared to other hybrid models, demonstrating superior predictive performance and robustness in PTB incidence forecasting.

**Table 4:** Comparison of the proposed hybrid model with the existing hybrid models.

Models	MAE	RMSE	MAPE
ARIMA+GRNN [16]	0.688	0.968	21.419
SARIMA+RNN [46]	0.667	1.079	19.879
SARIMA+GRNN [47]	0.726	1.108	21.146
SARIMA+BPNN [17]	1.486	1.873	47.727
SARIMA+SVR [19]	0.683	0.917	15.132
SARIMA+LSTM [13]	0.539	0.904	14.832
SARIMA+ETS [18]	0.740	1.087	19.376
SVR+BPNN [17]	1.134	1.466	32.658
**The proposed model**	**0.434**	**0.737**	**10.795**

### 3.6 Statistical analysis

To statistically validate the superiority of the proposed model, the Diebold-Mariano (DM) test was employed in this study. This statistical method is specifically designed to compare the predictive accuracy of two competing forecasting models [48]. It is widely used in time series analysis to determine whether the performance difference between a proposed model and a benchmark alternative is statistically significant.

The predictive accuracy of the proposed model was compared against each benchmark model in a pairwise manner using the DM test. This test assesses the null hypothesis of equal predictive accuracy. A significantly negative DM statistic indicates that the proposed model incurs a lower forecast loss, whereas a positive value favors the benchmark. As summarized in Table 5, the DM statistic is negative and significant (*p* < 0.05) in most comparisons, demonstrating that our model yields significantly smaller forecast errors than the alternatives over the 2017–2019 period.

**Table 5:** DM test statistics for comparing the proposed model and benchmark models.

Models	DM	*p* value	95% CI low	95% CI high
SARIMA	−1.003	0.316	−0.911	0.308
ARIMA	−1.153	0.249	−1.144	0.315
GM (1,1)	−4.475	7.64 × 10^−6^	−3.437	−1.292
ETS	−2.064	0.039	−1.979	−0.017
SVR	−1.988	0.046	−1.122	0.011
ELM	−4.654	3.24 × 10^−6^	−3.160	−1.241
XGBoost	−4.112	3.91 × 10^−5^	−4.775	−1.618
BPNN	−3.064	0.002	−3.145	−0.638
RNN	−3.677	2.36 × 10^−4^	−14.23	−4.109
LSTM	−4.014	5.95 × 10^−5^	−11.16	−3.665
ARIMA+GRNN	−2.930	0.003	−0.667	−0.121
SARIMA+RNN	−1.692	0.091	−1.364	0.123
SARIMA+GRNN	−1.752	0.079	−1.474	0.108
SARIMA+BPNN	−3.735	1.88 × 10^−4^	−4.577	−1.353
SARIMA+SVR	−2.069	0.038	−0.588	−0.005
SARIMA+LSTM	−1.245	0.213	−0.721	0.173
SARIMA+ETS	−2.347	0.018	−1.191	−0.086
SVR+BPNN	−3.429	0.001	−2.556	−0.655

For a small subset of benchmarks, the difference was not statistically significant (*p* ≥ 0.05). It is important to note that a non-significant result does not imply the benchmark is superior; rather, it suggests that the available sample of 36 monthly predictions may lack the statistical power to detect a modest but real difference, particularly in the presence of serial correlation in forecast errors. In conclusion, the results confirm that the proposed model significantly outperforms the majority of benchmark models, and the few non-significant cases are best interpreted as a consequence of limited sample size, autocorrelation, and heteroscedasticity, rather than as evidence against the model’s efficacy.

## 4. Results and discussion

This study developed a high-performance hybrid model for forecasting PTB incidence in Chongqing, China. The proposed SARIMA//SVR + ELM(SSA) model demonstrated superior performance by effectively integrating linear components captured by SARIMA, nonlinear residuals modeled by SVR, and a final integration step using the SSA-optimized ELM. The PTB incidence series exhibited a clear long-term decreasing trend and stable seasonal fluctuations. Moreover, incorporating spatial data from adjacent provinces significantly enhanced the model’s accuracy, corroborating the spatial spillover effect of PTB transmission. Statistical tests confirmed that the proposed hybrid model was significantly superior to most benchmark models, thereby affirming the reliability of its PTB incidence forecasts.

A notable temporal performance drift was observed during the model’s testing phase, characterized by a rise in the mean absolute error from 0.28 in 2017 to 0.59 in 2019. The most significant instance of this was an over-prediction for January 2019, where the forecasted incidence was substantially higher than the observed value (predicted: 6.79 vs. observed: 3.24 per 100,000). While this could be conventionally interpreted as a limitation in generalizability, we posit that it more constructively reflects a fundamental shift in the underlying trend of PTB incidence. This shift is likely attributable to the accelerated and enhanced interventions implemented under China’s “13th Five-Year” National TB Control Plan initiated in 2017 [49]. As the model was primarily trained on pre-2017 data, which captured a period of more gradual decline, it could not fully anticipate the steeper reduction in incidence driven by these structural policy breaks. From a public health planning perspective, this tendency for conservative, slightly over-estimating forecasts is not entirely detrimental, as it inherently creates a safety buffer for resource allocation, thereby enhancing the practical utility and decision-making robustness of the modeling framework.

In the current era dominated by data-intensive deep learning, our study yielded a counterintuitive finding: complex models like RNN and LSTM did not achieve superior performance. The root cause lies in the data-sparse nature of this study, where the 144 monthly data points with high variance were insufficient for such models to learn effectively, making them prone to overfitting and capturing noise rather than the underlying epidemiological pattern [50]. In this context, the ELM emerged as a particularly suitable choice. Its mechanism of using randomly assigned hidden layer weights and analytically calculating output weights grants it exceptional computational efficiency and remarkable resistance to overfitting on limited samples [51]. Complementing this, SVR was selected for its structural risk minimization principle, which provides inherent robustness against noise and outliers by constructing an ε-insensitive loss function [52]. Therefore, the ELM-SVR combination in our hybrid framework effectively balances computational efficiency, robustness, and nonlinear fitting capability, proving itself well-suited for medium-scale, volatile time-series forecasting tasks like the one in this study.

In the experiment investigating the impact of spatial features, we observed that forecasting performance generally improved as more adjacent provinces were included, although this trend was not strictly monotonic. For instance, performance with three provinces was slightly inferior to that with two, a common non-monotonic pattern in multivariate modeling attributable to complex feature interactions, partial redundancy, and the inherent bias-variance trade-off of increasing model dimensionality [53]. Epidemiologically, this suggests meaningful geographical heterogeneity in the TB epidemic across regions. While data from adjacent provinces overall provide valuable spatial spillover signals, the influence of each specific province is not uniform, potentially due to variations in population mobility, data reporting practices, or local intervention timelines. Consequently, information from a third province might partially conflict with or dilute existing signals until a more comprehensive spatial context is established with additional provinces. This finding underscores that indiscriminately adding data sources is suboptimal; future work should refine spatial feature integration by employing weighted matrices or mobility-based coupling indicators to build more precise and interpretable forecasting systems.

## 5. Conclusion and future perspectives

In summary, this study successfully developed an accurate and robust hybrid model for forecasting PTB incidence. Through a profound analysis of the model’s performance, we have not only validated its technical superiority but, more importantly, connected its behavior to real-world public health practice: the temporal variation in forecast error may reflect effectiveness of disease control that exceeded expectations; the data regime dictated that simpler, efficient models were more practical than complex ones; and the non-monotonic spatial pattern revealed the complex heterogeneity of disease spread. These discussions transcend mere model performance comparison and provide a new perspective for utilizing AI tools to understand and evaluate infectious disease control policies [54].

Future work will focus on integrating more real-time data streams (e.g., internet search indices, climate data) and finer-grained human mobility information to better capture the dynamic factors leading to structural breaks, thereby constructing more proactive and adaptive early warning systems for infectious diseases.

## Supporting information

S1 File PTB_code. (ZIP)

S1 Appendix The hyperparameter of the proposed model. (DOCX)
