# Supplementary material for: A hybrid machine learning model for pulmonary tuberculosis forecasting of Chongqing with adjacent-region data
Source: PLoS One. 2025 Dec 31;20(12):e0339453. doi: 10.1371/journal.pone.0339453 (PMC12755765; doi:10.1371/journal.pone.0339453)
Supplement: S1 Appendix — (DOCX) [file pone.0339453.s002.docx]

S2 Appendix. The hyperparameter of the proposed model.

| Hyperparameter | Value |
| --- | --- |
| SARIMA | |
| Non-seasonal order | (1, 1, 1) |
| Seasonal order | (0, 1, 1, 12) |
| SVR | |
| Lag length | 12 |
| kernel | rbf |
| Regularization parameter | 10 |
| Epsilon-insensitive loss margin | 0.1 |
| ELM | |
| Hidden layer size | 32 |
| Activation function | tanh |
| Ridge regression regularization | 0.001 |
| SSA | |
| Population size | 40 |
| Iterations | 100 |
| Producer ratio | 0.2 |
| Warning ratio | 0.1 |
